# The complete chloroplast genome sequence of *Rhododendron oreodoxa* var. *fargesii* (Ericaceae), an ornamental plant

**DOI:** 10.1080/23802359.2022.2093667

**Published:** 2022-07-08

**Authors:** Qingsong Zhu, Ying Cao, Yunong Yao, Yangyang Zhang

**Affiliations:** a School of Horticulture, Xinyang Agriculture and Forestry University, Xinyang, Henan, P. R. China; b College of Horticulture and Forestry Sciences, Huazhong Agricultural University, Wuhan, Hubei, P. R. China

**Keywords:** *Rhododendron oreodoxa* var. *fargesii*, plastid genome, phylogeny analysis, ericaceae

## Abstract

*Rhododendron oreodoxa* var. *fargesii* is an evergreen shrub or small tree, which has great ornamental and huge medicinal value. The complete chloroplast genome of this species has not been reported. In the present study, we obtained the chloroplast complete genome, the circular genome was 200,787 bp in total length, and 141 genes were identified, including 93 protein-coding, 40 tRNA genes, and 8 rRNA genes. The results of the phylogenetic analysis supported the idea that Rhododendron belongs to the Ericaceae family and that *R. oreodoxa* var. *fargesii* is closely related to *R. griersonianum* in the Ericaceae family. This study will provide useful data onto phylogeny research and genomic selective breeding of *R. oreodoxa* var. *fargesii.*

*Rhododendron oreodoxa* var. *fargesii* (Franch.) Chamb. ex Cullen et Chamb. species first published in Notes Roy. Bot. Gard. Edinburgh, 37:331 (1979) (https://powo.science.kew.org/) and it is a valuable ornamental plant. Their branches are shiny and hairless, and they are purple and bright in color during their first year of growth. They are hardy, evergreen, broad-leaved trees; with the development of cultivation techniques, there are now more than 900 listed *Rhododendron* species, as well as numerous hybrids (Vetaas 2002; Fiona et al. 2006). Some suitable environmental conditions can favor the growth of the *R. oreodoxa* var. *fargesii*. We used high-throughput sequencing technology that reported the complete chloroplast genome sequence of *R. oreodoxa* var. *fargesii* to provide genetic information for the construction of phylogenetic relationships among Ericaceae. Therefore, we enriched the genetic information and provided the foundation of knowledge for further studies of this species.

*Rhododendron oreodoxa* var. *fargesii* leaf specimens were sampled from Shennongjia National Park, Hubei Province, China (110°18′ E, 31°26 N; altitude: 2,858 m) and quickly dried with silica gel for DNA extraction. Afterwards, these specimens (code number:ZQS01) were stored in the refrigerator at −80 °C in the laboratory of the Horticultural Plant Biotechnology, Xinyang Agriculture and Forestry University (Resource person: Qingsong Zhu&Email: horticres@163.com). Plant material collection complied with the Convention on Biological Diversity and the Convention on the Trade in Endangered Species of Wild Fauna and Flora. DNA was extracted from leaf tissues, total genomic DNA was extracted from fresh leaves of *R. oreodoxa* var. *fargesii*.with Rapid Plant Genomic DNA Isolation Kit (BALB, Beijing, China), which were collected from Xinyang Agriculture and Forestry University, China. The DNA sample was sent to Shanghai Origingene Biotechnology Limited Company to construct a DNA library and was sequenced by using the Illumina NovaSeq 6000 sequencing platform (Illumina, San Diego, CA, USA). In addition, data processing was performed using the Illumina High-throughput sequencing platform (script filtering in NOVOPlasty) (Dierckxsens et al. 2017). Approximately 11.32 GB of raw data were generated with 150 bp paired-end read lengths. The complete plastid genome of *R. platypodum* (GenBank accession number: NC_053746) was chosen as the reference. The annotated genomic sequence has been submitted to GenBank (accession number: OL639014).

The plastid genome of *R. oreodoxa* var. *fargesii* was assembled by using the GetOrganelle pipeline (https://github.com/Kinggerm/GetOrganelle), and the reads were then viewed and edited by using Bandage (Wick et al. 2015). The cp genome annotation was assembled based on the comparison by Geneious v 11.1.5 (Biomatters Ltd, Auckland, New Zealand) (Kearse et al. 2012). The complete cp genome of *R. oreodoxa* var. *fargesii* was a circular shape of 200,787 bp in length, consisting of four distinct regions: a large single-copy (LSC) region of 108,429 bp, a small single-copy (SSC) region of 2,748 bp, and a pair of inverted repeats (IR) of 44,805 bp. The complete cp genome consisted of 141 genes, including 93 protein coding genes, 40 tRNA genes, 8 rRNA genes, and 41.57% GC content. Phylogenetic analyses, including five Ericaceae species, ten Styracaceae species, seven Primulaceae species, six Actinidiaceae species, two Pentaphylacaceae species, one Icacinaceae species, and one Eucommiaceae species, were performed by using complete cp genomes. All of them were downloaded from NCBI GenBank. The sequences were aligned via MAFFT v7.307 (Katoh and Standley 2013), and the phylogenetic tree was constructed by using MEGA X (Kumar et al. 2018). The phylogenetic tree revealed that *R. oreodoxa* var. *fargesi* was closely related to *R. griersonianum* with strong support (Figure 1).

**Figure 1.**
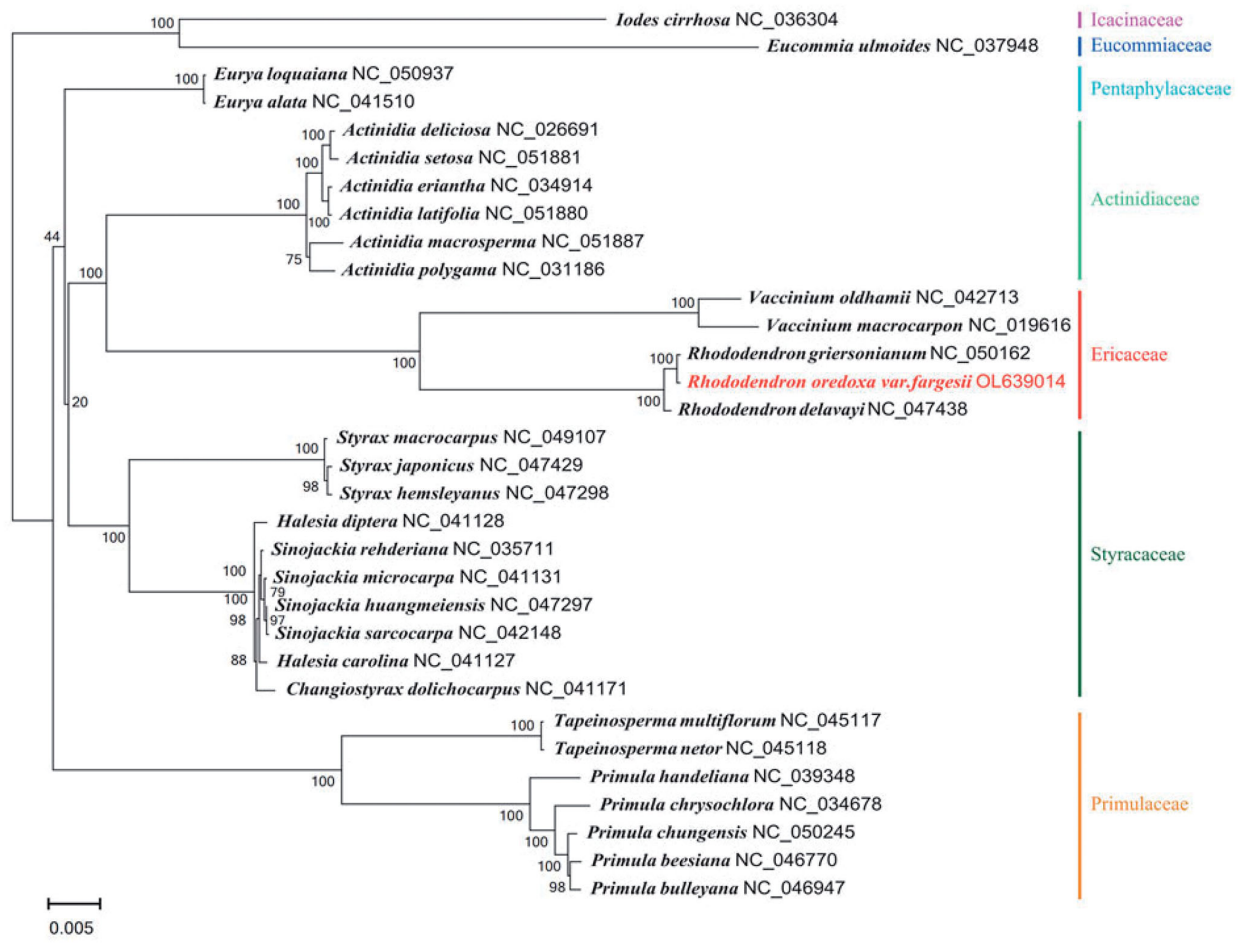
Maximum-likelihood phylogenetic tree based on complete cp genomes. Numbers close to each node are bootstrap support values.

## Data Availability

The genome sequence data that support the findings of this study are openly available in GenBank of NCBI at [https://www.ncbi.nlm.nih.gov/] under the accession OL639014. The associated BioProject, SRA, and Bio-Sample numbers are PRJNA781766, SRX13219942, and SAMN23288694, respectively.
